# Cross-condition and cross-platform remaining useful life estimation via adversarial-based domain adaptation

**DOI:** 10.1038/s41598-021-03835-2

**Published:** 2022-01-18

**Authors:** Dongdong Zhao, Feng Liu

**Affiliations:** School of Computer and Information Technology, Beijing Jiaotong University, Beijing, 100089 China

**Keywords:** Electrical and electronic engineering, Mechanical engineering

## Abstract

Supervised machine learning is a traditionally remaining useful life (RUL) estimation tool, which requires a lot of prior knowledge. For the situation lacking labeled data, supervised methods are invalid for the issue of domain shift in data distribution. In this paper, a adversarial-based domain adaptation (ADA) architecture with convolution neural networks (CNN) for RUL estimation of bearings under different conditions and platforms, referred to as ADACNN, is proposed. Specifically, ADACNN is trained in source labeled data and fine-tunes to similar target unlabeled data via an adversarial training and parameters shared mechanism. Besides a feature extractor and source domain regressive predictor, ADACNN also includes a domain classifier that tries to guide feature extractor find some domain-invariant features, which differents with traditional methods and belongs to a unsupervised learning in target domain, which has potential application value and far-reaching significance in academia. In addition, according to different first predictive time (FPT) detection mechanisms, we also explores the impact of different FPT detection mechanisms on RUL estimation performance. Finally, according to extensive experiments, the results of RUL estimation of bearing in cross-condition and cross-platform prove that ADACNN architecture has satisfactory generalization performance and great practical value in industry.

## Introduction

Remaining useful life (RUL) estimation, which is one facet of prognostics and health management (PHM) aiming to provide users with an integrated view about the health state of a machine or an whole system^1^, defined as “the length from the current time to the end of useful life”^2^. With the development of sensing technology, deep learning (DL) technology with nonlinear deep representation capabilities has gradually matured. A large amount of studies^3–9^ on RUL estimation of bearing in same operating condition have obtained good results.

Although every healthy mechanical component will has a rated working time after it is created, varying operating conditions and platforms will affect the service life of mechanical component to varying degrees. In the case where the training data and the test data come from different operating conditions and platforms, the generalization ability on the test data is usually poor.

In the case of varying operating conditions and platforms, there are many factors influencing on performance of RUL estimation, such as feature extractor and regressive predictor, but in the final analysis, it is caused by the number of observation samples, first predictive time (FPT) detection method, etc. For example, when migrating from a source bearing entity with observation samples *N*1 to a target bearing entity with observation samples *N*2, if *N*1 is much smaller than *N*2, the RUL predicted by the target domain usually fluctuates more strongly. We assume that the bearing entity degrades linearly after the failure prediction time point. The source bearing entity 1 has N1 samples, target bearing entity 1 has N2 samples, and their FPT are respectively FPT1 and FPT2. It is a challenge for feature extractors and regressive predictors to accurately predict RUL. Particularly, the feature extractor needs to obtain discriminative features, and the regressive predictor needs to map the discriminative features to actual RUL value as accurately as possible.

Though there are many effective domain adaptation (DA) methods for classification, but we practically found that few regressive DA methods. Jiang et al.^10^ suggested that the regression space is usually continuous on the contrary, for example, there is no clear decision boundary. In fact, the observation samples have limited, the RUL estimation is still in a large discrete space. Jiang et al.^10^ firstly proposed regressive DA for keypoint detection, which proved the effectiveness of DA in regressive prediction tasks. For RUL estimation of bearing under varying conditions or platforms, the key factor is how to assign cross-invariant features built by neural network corresponding same health status a equal estimated RUL, which is vital for unsupervised RUL estimation in cross-domain.

Therefore, we found that in fact, the idea of regressive DA is still the same as the classification algorithm, such as SVM trying to find margins of boundaries between different classes. RUL estimation under varying operating conditions or varying platforms aims to find a boundary to distinguish the different operating states of bearing entity. In whole, there are three key points worth paying attention to:

1. Domain-invariant features. $F(\{x_{RUL=0.5}^{s,i}\}\approx F(\{x_{RUL=0.5}^{t,j}\}$, *F*(*x*) is the feature representation of sample *x*, $\{x_{RUL=0.5}^{s,i}\}$ denotes the samples data, where RUL equals to 0.5, of the *i*-th source bearing entity. $\{x_{RUL=0.5}^{t,j}\}$ denotes the samples, where RUL equals 0.5, of the *j*-th target bearing entity.
2. Discriminative features. $F(\{x_{RUL=0.5}^{s,i}\}$ is not equal to or far from $F(\{x_{RUL=0.6}^{s,i}\}$, and $F(\{x_{RUL=0.5}^{t,j}\})$ is not equal to or far from $F(\{x_{RUL=0.6}^{t,j}\})$.
3. If the input of regressive predictor are some similar domain-invariant features respectively come from source domain and target domain, and the predicted results of regressive predictor should be approximately equal with each other. We use the formula to express as: $Y(F(\{x_{RUL=0.5}^{s,i}\})) \approx Y(F(\{x_{RUL=0.5}^{t,j}\}))$, where *Y*() is the function of regressive predictor.

Motivated by domain adaptation neural networks (DANN)^11^, in this paper, we introduce the original intention proposed by the DANN architecture into machinery RUL estimation. Through combining with the superiority of CNN in vibration signals process, adversarial-based domain adaptation (ADA), refers to ADACNN, consists of three parts: feature extractor, regressive predictor and domain classifier. Feature extractor firstly acquires domain-invariant and discriminative representation from raw vibration signals. The domain classifier, as an important auxiliary tool, forces feature extractor finding common space where samples have corresponding domain-invariant representation. The regressive predictor input a domain-invariant feature outputs a corresponding estimated RUL close to actual RUL value of the domain-invariant feature.

Overall, in this paper, we proposes a novel neural networks framework with ADA for RUL estimation of bearings in different condition and platform. The main contributions of this paper are as follows:

1. For scenarios lacking labels or no label, ADACNN can be simply switched for maximizing known labels’ value in real application scenarios. At the same time, it ensures excellent estimation accuracy in source domain and generalization ability in target domain. To our best knowledge, this framework with ADA is firstly introduced for RUL estimation of bearing in varying conditions and platform. In addition, in this paper, we just pay attention to a harder unsupervised case, that is, there are all unlabeled data in target domain.
2. The ADACNN was verified on two public datasets: cross-condition experimental scenarios and cross-platform experimental scenarios on FEMTO and XJTU-SY dataset introduced afterthis.
3. The proposed methodology compares with two non-adapted models respectively training with only source data and only target data to verify the generalization ability of proposed ADACNN.

## Preliminaries

Transfer learning (TL) was grouped into three classifications: inductive TL, transductive TL and unsupervised TL^12^. Given a source domain $D_{source}$ and a corresponding learning task $T_{source}$, a target domain $D_{target}$ and a corresponding learning task $T_{target}$. In^12^, DA belongs to transductive TL, that is, $T_{source}=T_{target}$ and $D_{source} \ne D_{target}$. DA is divided into three categories in^13^: discrepancy-based methods, adversarial-based methods and reconstruction-based methods. Among them, DA methods are the most popular. Discrepancy-based methods use source and target domain data to fine-tune the model to reduce domain shift. Discrepancy-based DA can be divided into the following according to the criteria used: class criterion, statistic criterion, architecture criterion and geometric criterion. Adversarial-based methods use domain classifier to encourage domain confusion through an adversarial objective. Specifically, a domain classifier tries to guide feature extractor to find a common space with domain-variant from source or target domain, until the classifier cannot distinguish whether the feature comes from source or target, the prediction (classification or regression) function of the source domain can be shared with target domain data. Reconstruction-based methods utilize data reconstruction as an auxiliary task to ensure feature invariance between domains (source and target)^11,12^. Obviously, DANN^11^ belongs to adversarial-based methods.

**Figure 1:**
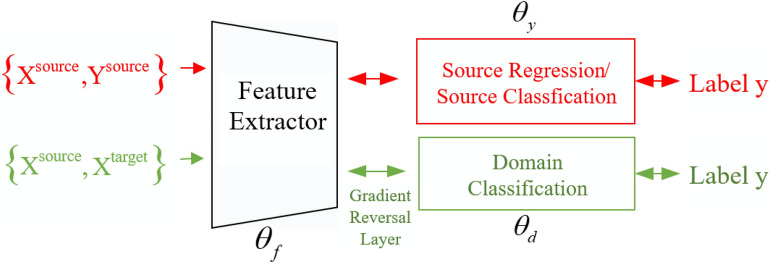
The architecture of DANN.

As shown in the Fig. 1, the red part is a supervised source training process, the green part is unsupervised learning phase in target domain, and black part represents feature extractor which is always based on CNN or RNN or their variants. The feature extractor is parametrised by $\theta _{f}$, prediction of main task (classification or regression that depends on main tasks) is parametrised by $\theta _{y}$, the domain classifier is parametrised by $\theta _{d}$. Of course, if the source domain is a supervised classification task in the adversarial model, then $\theta _{y}$ represents a classifier, and if it is a regression task, $\theta _{y}$ represents a regressive predictor. Source data with fully labeled and target data with no label are input into DANN. For the target data, it is an unsupervised learning process. The target data participate in the training of the $\theta _{d}$ to enhance the generalization ability of $\theta _{f}$. The target data labels are only used when participating in the evaluation during the test phase. We record source data as $(X^{source}, Y^{source})$ and target data as $(X^{target})$, and $D_{source} \ne D_{target}$ which means the distribution of source domain is different from the distribution of target domain. The $\theta _{f}$ is used to try to find a common space where no discriminative information between source domain data and target domain data, instead of maintaining its own characteristics in different domains. The source domain features extracted by $\theta _{f}$ are fed to $\theta _{y}$, then $\theta _{y}$ and $\theta _{f}$ are optimised by backpropragation. The target domain data features and source domain data features extracted by $\theta _{f}$ are fed to the $\theta _{d}$, then $\theta _{d}$ and $\theta _{f}$ are also optimised by backpropragation. The original aspiration is to minimize the loss of predictor (classifier and regressor) and maximize the loss of the domain classifier. After some iterative training, $\theta _{d}$ cannot distinguish whether the feature comes from the source domain or the target domain, we think that DANN has found a common feature space between source domain data and target domain data. By parameters sharing mechanism, the trained DANN can fine-tune to target domain data.

Therefore, from the perspective of theoretical realization, the composition of the loss function of DANN is divided into two parts: the prediction loss of the main task and the domain classification loss. The prediction loss $L_{y}^{i}$ of i-th batch examples is defined as the Eq. (1):

1 $$\begin{aligned} L_{y}^{i} = \min \limits _{\theta _{f}\theta _{d}}\left[ \frac{1}{N_{S}}\sum \limits _{i=1}^{N_S}L_{d}^{i}(\theta _{f},\theta _{y})+\alpha R(\theta _{f})\right] \end{aligned}$$

where $\alpha R(\theta _{f})$ is the regularisation factor, and it’s weight is $\alpha$. Inspired by proxy distance, the optimisation problem of $\theta _{d}$ is denoted as the Eq. (2):

2 $$\begin{aligned} R(\theta _{f}) = \max \limits _{\theta _{d}}\left[ -\frac{1}{N_{S}}\sum \limits _{i=1}^{N_S}L_{d}^{i}(\theta _{f},\theta _{d})-\frac{1}{N_{T}}\sum \limits _{i=1}^{N_T}L_{d}^{i}(\theta _{f},\theta _{d})\right] \end{aligned}$$

Equations (1) and (2) are made up of a min–max adversarial optimisation procedure. DANN includes a deep feature extractor (black box in Fig. 1) and a deep label predictor (red box in Fig. 1), which together construct a traditional standard feedforward architecture. The domain classifier (green box in Fig. 1) is connected to the feature extractor. Last but not least, gradient reversal layer (GRL) plays an indispensable role in DANN, which builds a bridge between feature extractor and classifier for guiding feature extractor to acquire domain-invariant features. In the updation processes of model parameters by back-propagation, the gradient is multiplied by a certain negative constant through GRL.

In short, the training processes of DANN are constraint through the min–max formula (Eqs. 1 and 2), and stopped training when the optimal balance train-off is found.

## Proposed method

### Problem formulation

In this paper, we proposed a framework with ADA for RUL estimation, which constitutes feature extractor, domain classifier, and a regressive predictor. It should be pointed out that we assume the source domain training are run-to-failure vibration data. Let $X^{source} =\{x_{i}^{s,j}\}_{i=1}^{n^{s,j}}$, $j=1, 2, \ldots , N_{source}$, represents the whole source sample data, $N_{source}$ is the number of the source bearing entity. $x_{i}^{s,j}$ denotes the *i*-th sample of the *j*-th bearing entity in source domain, where $n^{s,j}$ is the number of samples for the *j*-th bearing entity in source domain. By analogy, $X^{target} =\{x_{i}^{t,j}\}_{i=1}^{n^{t,j}}$, $j=1, 2, \ldots , N_{target}$, represents the whole target sample data, $N_{target}$ is the number of the target bearing entity. $x_{i}^{t,j}$ denotes the *i*-th sample of the *j*-th bearing entity in target domain, where $n^{t,j}$ is the number of samples for the *j*-th bearing entity in target domain.

**Figure 2:**
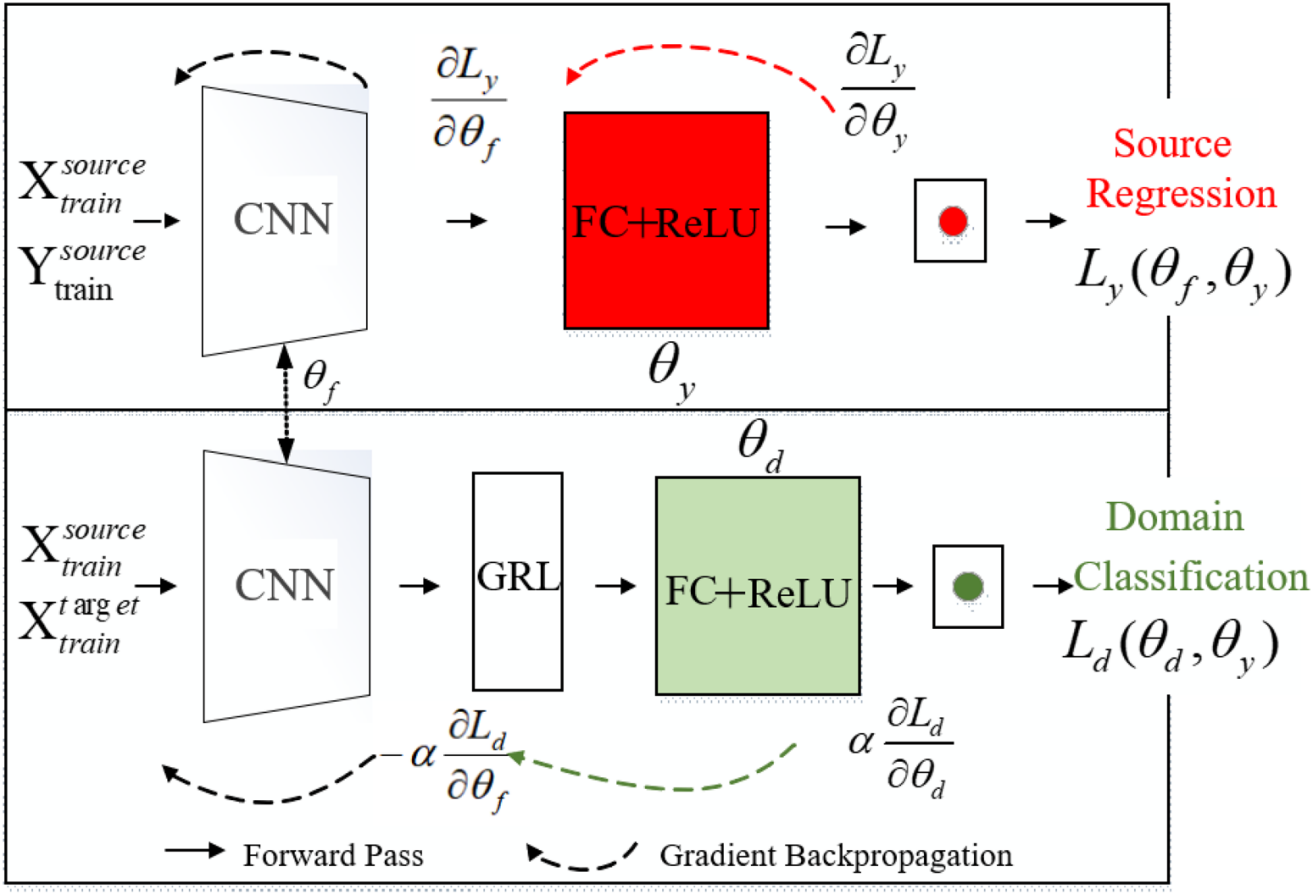
The architecture of ADACNN.

The methodology proposed in this paper mainly includes two steps as follows:

1. Data preparation. Calculate RUL percentage label $Y^{source}=\{y_{i}^{s,j}\}_{i=1}^{n^{s,j}}$ corresponding to the *i*-th sample of the *j*-th bearing entity in source domain.
2. Building and training the ADACNN model. In addition to $X^{source}$ and $Y^{source}$ in source domain as input of ADACNN, unlabeled data $X^{target}$ in target domain are also used as input when training ADACNN, which includes three parts shown in Fig. 2:
  - A regressive predictor parameterized by $\theta _{y}$ is introduced to accomplish main regression task through a supervised learning in source domain.
  - A feature extractor parameterized by $\theta _{f}$ finds a common space with domain-variant feature from source and target domain data.
  - A domain classifier parameterized by $\theta _{d}$ combined with GRL can not make the data output by the feature extractor be distinguished.

### Experimental design

#### Data preparation

The source domain data are divided into training data ($X_{train}^{source}$, $Y_{train}^{source}$) and test data ($X_{test}^{source}$, $Y_{test}^{source}$). The target domain data are also divided into training data $X_{train}^{target}$ and test data ($X_{test}^{target}$, $Y_{test}^{target}$). $X_{train}^{source}$ are used when training the ADACNN. $X_{test}^{source}$ are used as input for observing the maturity of model training. $X_{train}^{target}$ participates in unsupervised training ADACNN to improve its generalization ability, $X_{test}^{target}$ involves in the evaluation of ADACNN. It should be note that the training data are the run-to-failure data, and the test data are truncated data.

The training label generation: taking FPT as the boundary, the actual RUL values of data samples before FPT point are equal to 1, and the actual RUL value corresponding to the *j*-th sample after FPT point drops from 1 to 0 successively, which denoted as $\frac{n_{i}^{j}}{Total_{i}-FPT_{i}}$, where $n_{i}^{j}$ represents the *j*-th sample after FPT point of *i*-th bearing entity, $Total_{i}$ is equal to the number of whole run-to-failure samples of *i*-th bearing entity, and $FPT_{i}$ represents the number of samples before FPT point of *i*-th bearing entity.

The test label generation: $RUL_{i}=1-\frac{K}{Total_{i}-FPT_{i}}$. $FPT_{i}$ represents the FPT of *i*-th bearing entity. We assume that known truncation points are always after FPT point, and *K* denotes the number of samples between truncation point and FPT point is *K*.

Usually, before FPT point, it is not necessary or difficult to predict RUL because there is no obvious sign of degradation, and after this point, signs of degradation began to appear. Therefore, a FPT detection mechanism is very important to capture real-time changes. Kurtosis^3,14,15^ was used to detect FPT. Many feature-fused methods combine time domain with frequency domain vibration characteristics. References^16,17^ fused some features into one index descripting degradation process, and it is worth mentioning that these features are obtained by calculating the mahalanobis distance (MD) from original healthy state, which is a relative feature and suitable for some scenarios of vibration signal processing. In this paper, we will explore the impact of different FPT detection methods on RUL estimation.

#### Data normalization

In order to speed up the training speed and align the test data with the training data during the test, we have some pre-processing for the vibration data. Data normalization mainly includes four parts:

$$norm(X_{test}^{source},X_{train}^{source}),\quad norm(X_{train}^{source}, X_{train}^{source}),$$

and

$$norm(X_{test}^{target},X_{train}^{target}),\quad norm(X_{train}^{target}, X_{train}^{target}).$$

where *norm*(*a*, *b*) represents the normalization function and consists of two steps: firstly to calculate the mean *m* and variance *d* of *b*, and secondly to normalize *a* by *m* and *d*.

#### Building the ADACNN

1. Initialize feature extractor: The input parameters of feature extractor include input data, number of CNN layers, number of filters per layer, and dropout rate. The kernel size of first layer equals to 25 by default (Has been proven its effectiveness in^18^), and the remaining layers are initialized according to input parameters. The feature extractor mainly includes one dimensional convolution layer (Conv1D), activation layer, Dropout, and MaxPooling1D. The output of feature extractor are latent features, its dimension depends on the initialization parameters *f*.
2. Initialize regressive predictor: The input parameters of regressive predictor include input data, number of convolution network layers, and number of nodes per layer. The architecture of regressive predictor mainly consist of FCN, activation layer, and dropout layer. Finally, the output predicted value is between 0 and 1, indicating RUL percentage. The closer the predicted value is to 1, the healthier it is, and the closer it is to 0, the closer it is to a fault.
3. Initialize classifier: The classifier includes FCN, Activation layer, Dropout layer.
4. Construct regression model: Source regression model consists of feature extractor and regressive predictor. The output latent features of the feature extractor is fed to the regressive predictor after passing through a flatten layer.
5. Construct domain classification model: Through the parameter sharing mechanism of feature extractor, domain classification model mainly consists of feature extractor, GRL, and domain classifier.

#### Training the ADACNN

**Figure 3:**
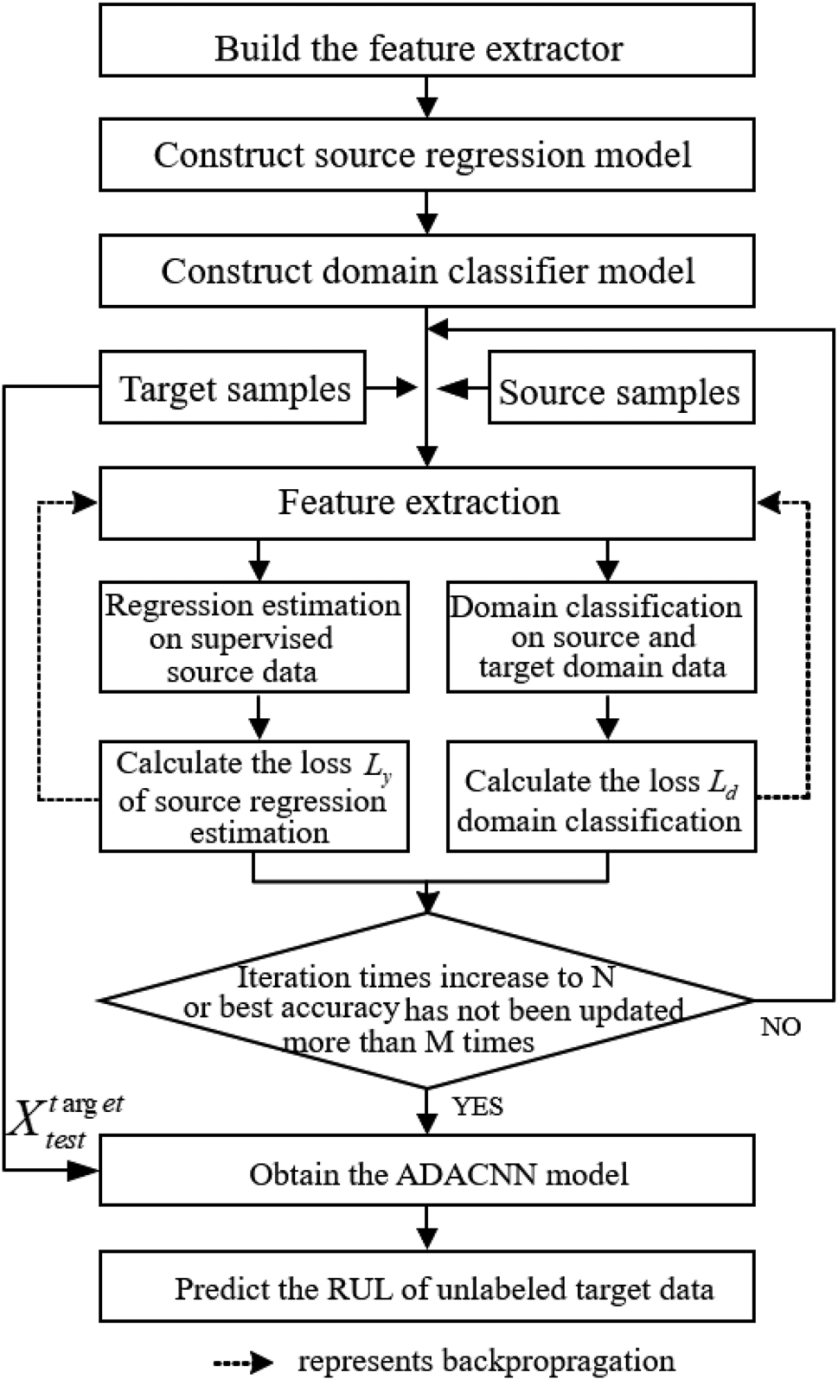
The schematic diagram of the process of the proposed model.

1. Initialization: Start iterative training with iteration $i=0$. Patience value M = 0. In order to reduce the memory pressure, data are read in batches in each iteration.
2. Training source regressive predictor and domain classifier: As shown in Fig. 2, through the forward propagation mechanism, the source regression model takes $X_{train}^{source}$ and $Y_{train}^{source}$ as input and RUL prediction value as output, according to the known $Y_{train}^ {source}$ to calculate the prediction loss. The domain classifier model takes $X_{train}^{source}$ and $X_{train}^{target}$ as input, outputs binary classification and calculate the loss of domain classification, and then update the parameters of the regressive predictor and the feature extractor and classifier through the backward propagation method of gradient learning as shown in Fig. 2. The *i*-th updation formula are defined as Eq. (3):
  3 $$\begin{aligned} \begin{aligned} \theta _{f}&=\theta _{f}-\mu \left( \frac{\partial L_{y}^{i}}{\partial \theta _{f}}-\alpha \frac{\partial L_{d}^{i}}{\partial \theta _{f}}\right) \\ \theta _{y}&=\theta _{y}-\mu \left( \frac{\partial L_{y}^{i}}{\partial \theta _{y}} \right) \\ \theta _{d}&=\theta _{d}-\mu \left( \frac{\partial L_{d}^{i}}{\partial \theta _{d}} \right) \end{aligned} \end{aligned}$$.
3. Evaluate the ADACNN model by calculating the loss of RUL estimation: Calculate the accuracy of current model using $X_{test}^{source}$ and $Y_{test}^{source}$ by root mean square error (RMSE) evaluation metrics. If $RMSE_{i}$ is less best accuracy $RMSE_{best}$, then $RMSE_{i}$ will be assigned to $RMSE_{best}$, otherwise, the patience value M is increased by 1.
4. Judgement 1: If M is greater than the preset value, stop iterative training and save the model parameters of the *i*-th iteration.
5. Judgement 2: If iteration *i* increases to threshold, stop iterative training and save the result of the *i*-th iteration.
6. Start a new iteration: The entire experimental flow chart is shown in Fig. 3, the value of *i* plus one is re-assigned to *i*, and continue going to the step 2.
7. Testing the ADACNN: Use target test data $X_{test}^{target}$ to evaluate the accuracy of the trained model.

### Experimental setup

#### Dataset description

1. FEMTO dataset. The FEMTO dataset comes from an experimental platform called PRONOSTIA, where bearings’ degradation experiments are allowed to conduct in only few hours, this platform can obtain true bearing degradation data by accelerating bearing degradation under different operating conditions so that some data-driven techniques are studied further. PRONOSTIA includes three main parts: a rotating part, a degradation generation part, and a measurement part. For more rotating part and degradation generation, please refer to^19^. For the measurement part, there are two types of signals: temperature and vibration with horizontal and vertical respectively from their own acceleration sensor and temperature sensor. The algorithm proposed in this paper only uses vibration signals, and the sampling frequency of the acceleration sensor is 25.6 kHz. As tabulated in Table 1. FEMTO data set includes three different operating conditions, we use A to represent the FEMTO dataset, and Ai-j to represent the *j*-th bearing of *i*-th conditions in FEMTO. Six bearings data are run-to-failure data, which we use as training data. The 11 bearing data are truncated to predict the remaining life, which we use as test data.

**Table 1:** The information of FEMTO dataset.

Operating conditions	Training data	Test data
A1 (1800 rpm and 4000 N)	A1-1 $\sim$ A1-2	A1-3 $\sim$ A1-7
A2 (1650 rpm and 4200 N)	A2-1 $\sim$ A2-2	A2-3 $\sim$ A2-7
A3 (1500 rpm and 5000 N)	A3-1 $\sim$ A3-2	A3-3
2. XJTU-SY dataset. The XJTU-SY dataset was collected by the Xi’an Jiaotong university and the Changxing Sumyoung Techonology Company^20^. 32768 data points are collected on 1.28 s of every minute with sampling rate of 25.6 kHz. The tested processes of bearing are stopped when the amplitude of the vibration signal is higher than 20 g for protecting the test bed. There are two PCB 352C33 accelerometers are placed on the housing of the tested bearings, which are respectively on the vertical and horizontal axis. The information of XJTU-SY dataset are shown in Table 2.

**Table 2:** The information of XJTU-SY dataset.

Operating conditions	Training data	Test data
B1 (2100 rpm and 12 kN)	B1-1 $\sim$ B1-2	B1-3 $\sim$ B1-5
B2 (2250 rpm and 11 kN)	B2-1 $\sim$ B2-2	B2-3 $\sim$ B2-5

#### Data preprocessing

For FEMTO, 2560 data points are collected on 0.1 s of every 10 s with sampling frequency of 25.6 kHz. For XJTU-SY, 32768 data points are collected on 1.28 s of every minute with sampling rate of 25.6 kHz. In this study, we reduced the dimensions of the sample data of the two datasets to 1280 by way of dimensionality reduction. For FEMTO, we extract 0.05 s of data from every 0.1 s sample data. In other words, we reduce the dimensionality by half. For XJTU-SY, we extract 0.05 s of data from every 1.28 s of sample data. In other words, we reduce the dimensionality of sample data by 25.6 times.

#### Comparative methods

1. Comparison against methods with different FPT mechanisms. For FPT detection mechanism influences the performance on RUL estimation, we choose three FPT detection methods: MD, kurtosis and no FPT. MD and Kurtosis sensitive to early failure has been widely used in FPT detection. No FPT detection mechanism means that the bearing will degenerates from the initial state. We denotes these three methods as MD-ADACNN, Kur-ADACNN and NoFPT-ADACNN respectively.
2. Comparison against non-adapted methods. In order to verify whether the proposed ADA method works, we use the following two baselines for comparison. The Source-Only method is trained on the source domain data $X_{train}^{source}$ and tested in the target domain $X_{test}^{target}$. The Target-Only method is trained on the target domain data $X_{train}^{target}$ and tested on the target domain data $X_{test}^{target}$ (There is no intersection between training data and test data). To be fair, the parameters of feature extractor and regressive predictor of the Source-Only and Target-Only methods are consistent with the parameters of ADACNN.

### Implementation details

#### Evaluation metrics

Root mean square error (RMSE), and Score are used as performance metrics to evaluate the error between the predicted RUL and the true RUL. RMSE has been used in many publications^21–23^, and its definition formula is the Eq. (4)

4 $$\begin{aligned} RMSE = \sqrt{\frac{1}{K}\sum \limits _{i=1}^{K}(y_{i}-{\hat{y}}_{i})^{2}}, \end{aligned}$$

where ${\hat{y}}_{i}$ and $y_{i}$ is the predicted RUL and actual RUL of *i*-th test sample, *K* denotes the total number of test samples. A larger RMSE value means a larger prediction error.

Score, defined as Eq. (5), used in this study was first proposed in^19^ and has been used in many studies^3,22,23^. The predicted RUL is greater than or less than the actual RUL should be treated differently. In other words, in the case of the same absolute value, the penalty for a positive value is less than the penalty for a negative value

5a $$\begin{aligned} Score = \sum \limits _{i=1}^{K}s_{i} \end{aligned}$$

5b $$\begin{aligned} s_{i} = \left\{ \begin{array}{ll} e^{-\frac{err_{i}}{13}}-1, &{} err_{i}\ge 0\\ e^{\frac{err_{i}}{10}}-1, &{} err_{i}< 0 \end{array} \right. \end{aligned}$$

5c $$\begin{aligned} err_{i} = y-{\hat{y}}, \end{aligned}$$

where *K* is the total number of test samples. $y_{i}$, $\hat{y_{i}}$, and $err_{i}$ respectively represent actual RUL, predicted RUL and the difference between actual RUL and predicted RUL for the *i*-th testing data sample.

#### Hyper-parameter selection

In adaptation training processes, the learning rate of source domain regression and domain classification and the parameter of CNN largely determine the experimental performance. Therefore, we use the grid-search method to find the optimal learning rate ($\lambda _{y}$, $\lambda _{d}$, [Layer, units, Dropout]), and then manually fine-tune other parameters presented in Table 3. Overall, we did 6 cross-condition experiments (E1–E6) on the FEMTO dataset, 2 cross-condition experiments (E7–E8) on the XJTU-SY dataset, and 12 cross-platform experiments (E9–E20) on the FEMTO and XJTU-SY. Their parameter pairs are tabulated in Table 4.

**Table 3:** Hyperparameter evaluated in the proposed method.

Hyper-parameter	Range
Layers: ($\theta _{f}$, $\theta _{y}$, $\theta _{d}$)	$\{$1, 2$\}$
Units: ($\theta _{f}$, *f*, $\theta _{y}$, $\theta _{d}$)	$\{$16, 32, 64, 128, 256, 512$\}$
Learning rate: ($\theta _{y}$ ($\lambda _{y}$), $\theta _{d}$ ($\lambda _{d}$))	$\{$0.0001, 0.001, 0.01$\}$
Dropout rate	$\{$0.1, 0.3, 0.5, 0.7, 0.9$\}$
$\alpha$	$\{$0.8, 1.0, 2.0$\}$
Batch size	$\{$64, 128, 256$\}$
Threshold of patience M	$\{$20$\}$
Max iteration N	$\{$200$\}$

**Table 4:** Selected hyperparemeter for each source-target experiment pair.

No.	From to	CNN: Layer, (units), [Dropout]	*f*	Source regression: Layers, (units), [Dropout]	Domain classification: Layers, (units), [Dropout]	$\alpha$	Batch size	($\lambda _{y}$, $\lambda _{d}$)
E1	A1 $\xrightarrow []{}$ A2	2, (128, 64), 0.9	512	1, (64), 0.1	2, (256, 128), 0.9	2	256	0.01, 0.01
E2	A1 $\xrightarrow []{}$ A3	2, (128, 32), 0.5	64	2, (32, 16), 0.3	2, (32, 16), 0.3	0.8	256	0.0001, 0.0001
E3	A2 $\xrightarrow []{}$ A1	2, (64, 32), 0.1	64	2, (32, 32), 0.1	2, (16, 16), 0.1	1	256	0.001, 0.001
E4	A2 $\xrightarrow []{}$ A3	2, (128, 64), 0.1	512	2, (64, 32), 0.1	2, (64,32), 0.1	2	256	0.001,0.001
E5	A3 $\xrightarrow []{}$ A1	2, (64,32), 0.3	128	2, (64, 64), 0.1	2, (64, 64), 0.1	2	256	0.001, 0.001
E6	A3 $\xrightarrow []{}$ A2	2, (128, 32), 0.9	512	2, (128, 128), 0.1	2, (128, 64), 0.1	2	256	0.001, 0.001
E7	B1 $\xrightarrow []{}$ B2	2, (128, 32), 0.1	64	2, (256, 128), 0.1	2, (128, 64), 0.5	0.8	128	0.001, 0.001
E8	B2 $\xrightarrow []{}$ B1	2, (128, 32), 0.9	64	2, (256, 128), 0.1	2, (128, 64), 0.9	0.8	128	0.001, 0.001
E9	A1 $\xrightarrow []{}$ B1	2, (32, 32), 0.1	64	1, (64), 0.1	1, (64), 0.5	0.8	64	0.001, 0.001
E10	A1 $\xrightarrow []{}$ B2	2, (128, 32), 0.1	64	1, (64), 0.1	2, (64, 64), 0.5	0.8	64	0.001, 0.001
E11	A2 $\xrightarrow []{}$ B1	2, (32, 32), 0.1	64	1, (64), 0.1	1, (64), 0.5	0.8	64	0.001, 0.001
E12	A2 $\xrightarrow []{}$ B2	2, (32, 32), 0.1	64	1, (64), 0.1	1, (64), 0.5	0.8	64	0.001, 0.001
E13	A3 $\xrightarrow []{}$ B1	2, (32, 32), 0.1	64	1, (64), 0.1	1, (64), 0.5	0.8	64	0.001, 0.001
E14	A3 $\xrightarrow []{}$ B2	2, (32, 32), 0.1	64	1, (64), 0.1	1, (64), 0.5	0.8	64	0.001, 0.001
E15	B1 $\xrightarrow []{}$ A1	2, (32,32), 0.9	64	1, (64), 0.9	2, (64,64), 0.5	0.8	64	0.001,0.001
E16	B1 $\xrightarrow []{}$ A2	2, (128, 32), 0.9	64	1, (64), 0.9	2, (64, 64), 0.5	0.8	64	0.001, 0.001
E17	B1 $\xrightarrow []{}$ A3	2, (128, 32), 0.9	64	1, (64), 0.9	2, (64, 64), 0.5	0.8	64	0.001, 0.001
E18	B2 $\xrightarrow []{}$ A1	2, (64,64), 0.1	64	1, (32), 0.9	2, (64,64), 0.5	1	64	0.001,0.001
E19	B2 $\xrightarrow []{}$ A2	2, (128, 32), 0.9	64	1, (64), 0.9	2, (64, 64), 0.5	2	64	0.001, 0.001
E20	B2 $\xrightarrow []{}$ A3	2, (128, 32), 0.9	64	1, (64), 0.1	2, (64, 64), 0.5	0.8	64	0.001, 0.001

## Discussion

It should be pointed out that all the pictures in the following content are generated by MATLAB software based on experimental data.

### Cross-condition

**Figure 4:**
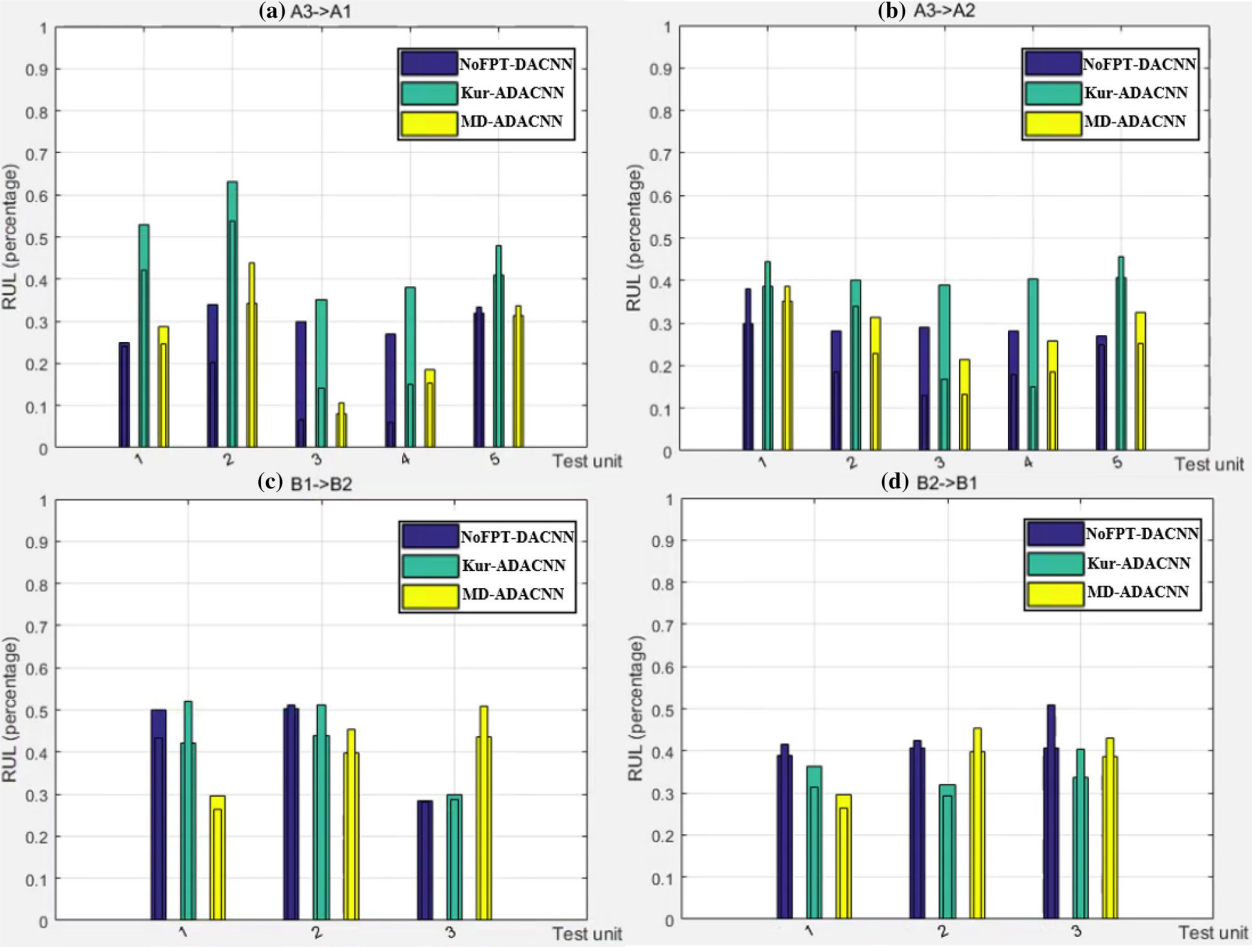
RUL estimation comparisons with different FPT detection mechanisms on FEMTO and XJTU-SY datasets: (**a**) A3 $\xrightarrow []{}$ A1, (**b**) A3 $\xrightarrow []{}$ A2, (**c**) B1 $\xrightarrow []{}$ B2, (**d**) B2 $\xrightarrow []{}$ B1.

In Fig. 4, the horizontal axis represents every test units of same operating condition in target domain (5, 5 and 1 on FEMTO, 3 and 3 on XJTU-SY), and the vertical axis represents RUL percentage. The thick histogram and the thin histogram respectively represent the predicted value and label of the same method. The closer the highest points of the thick histogram and the thin histogram are, the higher the accuracy. As described in Fig. 4, it can be seen that no matter which data set it is verified on, the predicted value of MD-ADACNN method is closer to its actual label. In addition, the MD-ADACNN method usually gives a predicted value slightly smaller than the true value, which will provide constructive warnings for engineering operation and maintenance engineers. However, for the other two FPT detection mechanisms, the prediction accuracy of the Kur-ADACNN method is obviously the lowest. The kurtosis-based FPT detection mechanism is an indicator in the vibration data, and the MD-based FPT detection mechanism is a relatively joint indicator of multiple indicators in the vibration data after dimensionality reduction in a relatively healthy state. Therefore, it can be seen from the results that the MD-based method is a more suitable FPT detection mechanism which is closer to the bearing degradation trend. It is worth emphasizing that the impact of FPT is only effective for experimental data sets, and does not mean that a specific FPT mechanism on all data sets can always maintain the best performance. Of course, the experimental work done can provide a certain degree of reference for the RUL prediction research of bearings from specific operating conditions or platforms to similarly configured operating conditions or platforms (from E1 to E20).

**Figure 5:**
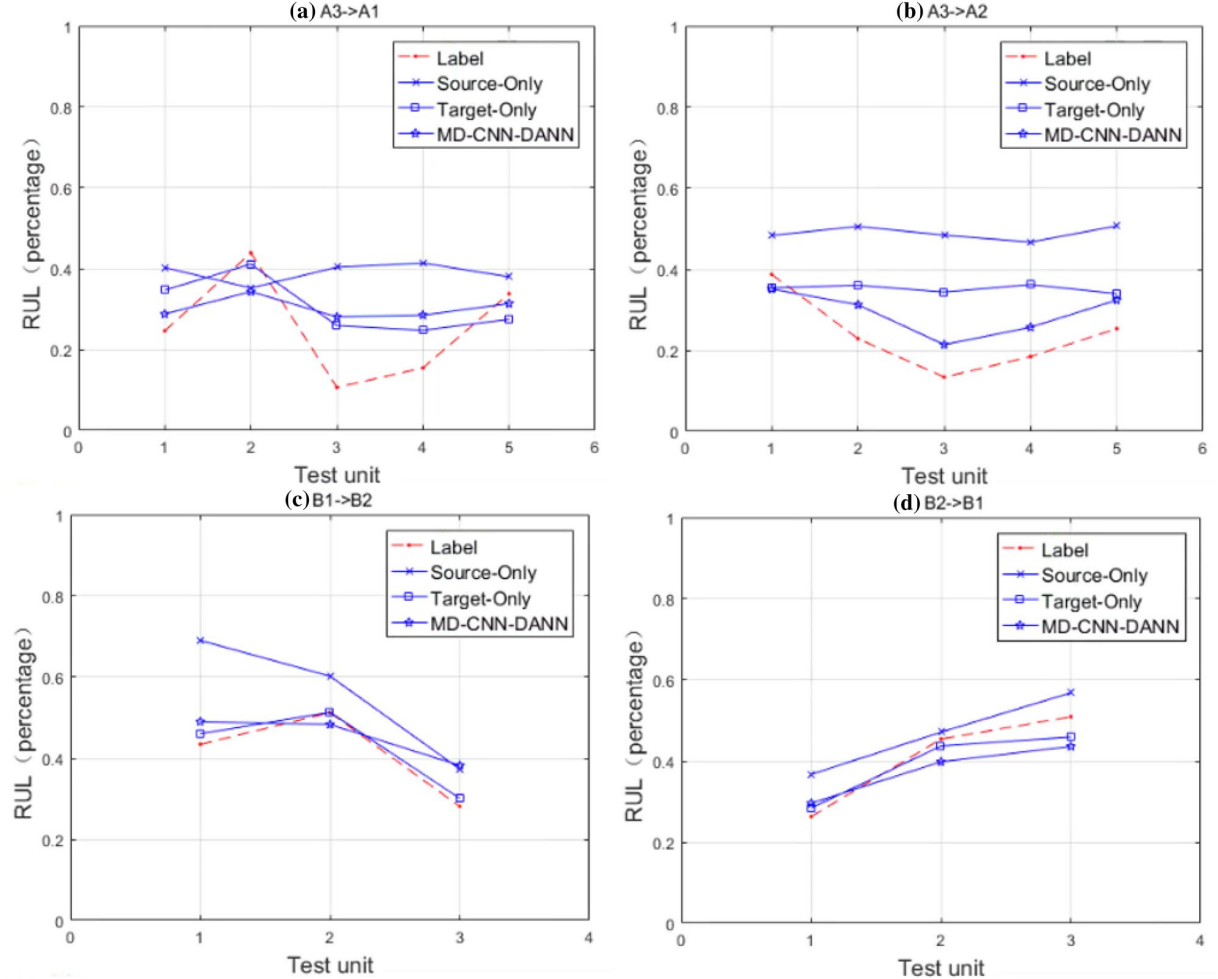
RUL estimation comparisons with source-only and target-only methods on FEMTO and XJTU-SY datasets. (**a**) E5: A3 $\xrightarrow []{}$A1, (**b**) E6: A3 $\xrightarrow []{}$ A2, (**c**) E7: B1 $\xrightarrow []{}$B2, (**d**) E8: B2 $\xrightarrow []{}$ B1.

From Fig. 5, we find that the RUL estimation results of MD-ADACNN method are between the results of Source-Only and Target-Only methods, and even more closer to the actual RUL value than Target-Only method (Fig. 5b), which proves effectiveness of ADA.

From the perspective of the FPT detection mechanisms, as shown in the cross-condition results of the three methods (MD-ADACNN, NoFPT-ADACNN and Kur-ADACNN) on the FEMTO dataset listed from E1 to E6 in Table 5, MD-ADACNN has obtained the four best RMSE and Score accuracy (E1: A1 $\xrightarrow []{}$A2, E3: A2 $\xrightarrow []{}$ A1, E5: A3 $\xrightarrow []{}$ A1 and E6: A3 $\xrightarrow []{}$ A2). In the other two cross-condition (A1 $\xrightarrow []{}$ A3 and A2 $\xrightarrow []{}$ A3), although MD-ADACNN has a larger error than the other two methods, from the 6 cross-condition experiments as a whole, MD-ADACNN predicts RUL more stable, and the two methods NoFPT-ADACNN and Kur-ADACNN, especially Kur-ADACNN, are greatly affected by the total number of cycle of units under varying conditions. In the FEMTO data set, the cycle of units under condition A3 is shorter, and the cycle of units under condition A1 and A2 is longer. Therefore, these two methods have better results in RUL estimation of cross-condition bearings from longer to shorter cycles, but very poor in the opposite case.

**Table 5:** RMSE/score ± standard deviation comparison between source-only, target-only, NoFPT-ADACNN, Kur-ADACNN and MD-ADACNN on FEMTO dataset and XJTU-SY dataset.

No.	Source-only		Target-only		MD-ADACNN		NoFPT-ADACNN		Kur-ADACNN	
	RMSE	Score	RMSE	Score	RMSE	Score	RMSE	Score	RMSE	Score
E1	53.2	101.3	32.6	64.6	36.2 ± 1.6	74.0 ± 46.4	41.5 ± 2.7	91.9 ± 66.7	36.5 ± 2.5	88.3 ± 88.2
E2	26.0	12.5	8.7	1.0	8.5 ± 14.4	5.1 ± 14.0	3.4 ± 4.0	0.3 ± 0.4	2.9 ± 0.5	0.3 ± 0.1
E3	39.4	239.8	22.5	15.3	26.8 ± 5.9	14.4 ± 10.9	44.2 ± 7.4	328.0 ± 700.0	64.3 ± 8.9	8542.5 ± 9330.2
E4	30.5	20.2	24.6	10.7	19.8 ± 18.8	10.3 ± 4.5	9.9 ± 9.1	1.9 ± 2.8	3.8 ± 1.3	0.4 ± 0.1
E5	67.0	2084.7	33.5	930.4	31.4 ± 3.2	7.1 ± 4.7	40.0 ± 7.4	136.6 ± 215.9	52.0 ± 4.0	544.4 ± 322.3
E6	48.8	1752.6	30.7	35.9	35.4 ± 0.8	37.2 ± 9.1	43.6 ± 14.6	1088.6 ± 2835.6	37.3 ± 2.0	89.4 ± 43.4
E7	18.3	10.1	13.0	1.0	13.3 ± 0.3	1.2 ± 0.6	14.0 ± 2.9	2.1 ± 1.1	17.6 ± 3.4	9.6 ± 1.0
E8	5.6	0.7	3.1	0.3	3.6 ± 0.7	0.4 ± 0.4	5.3 ± 2.2	0.6 ± 0.3	4.1 ± 0.8	0.7 ± 0.1

From the perspective of whether to use domain adaption technology in Table 5, in most experiment with cross-condition (E1: A1 $\xrightarrow []{}$ A2, E2: A1 $\xrightarrow []{}$ A3, E3: A2 $\xrightarrow []{}$ A1, E5: A3 $\xrightarrow []{}$ A1 and E6: A3 $\xrightarrow []{}$ A2), RMSE(Source-Only) > RMSE(MD-ADACNN) > RMSE(Target-Only). In these cross-condition (E2: A1 $\xrightarrow []{}$ A3 and E4: A2 $\xrightarrow []{}$ A3), RMSE(MD-ADACNN) RMSE(Target-Only), which shows that the generalization ability of ADA technology from condition A2 with a long cycle time to condition A3 with a short cycle time exceeds supervised algorithms that only use target data, that is, to some extent, the data in target domain are benefit to guide the whole ADA model fine-tune to target data.

Judging from the results of the five methods (Source-Only, Target-Only, MD-ADACNN, NoFPT-ADACNN and Kur-ADACNN in Table 5) verified on the FEMTO data set, basically, we found the effects of these three methods (MD-ADACNN, NoFPT-ADACNN and Kur-ADACNN) are superior to that of Source-Only, so these results prove that the validity of domain adaption technology in the research field of cross-condition bearing RUL estimation. Observing the results listed from E7 to E8 in Table 5. on the XJTU-SY data set from the same perspective, the same conclusion was confirmed again.

### Cross-platform

In order to further verify the performance of the proposed ADACNN method in the inter-platform domain for RUL estimation, we choose the three conditions in the FEMTO dataset as the source domain or target domain data, and at the same time, 2 conditions in the XJTU-SY dataset are used as target domain or source domain data, so there are a total of 12 experiments (E9–E20 tabulated in Table 6 between two platforms. Different from the cross-condition, the cross-platform experiment part only explores the research on the remaining life of different FPT establishment mechanisms under the same DA technology, because the superiority of the DA technology has been clearly proved in the previous experiments.

**Table 6:** RMSE/score ± standard deviation comparison under cross-platform on FEMTO and XJTU-SY.

No.	From to	MD-ADACNN		NoFPT-ADACNNs		Kur-ADACNN	
		RMSE	Score	RMSE	Score	RMSE	Score
E9	A1 $\xrightarrow []{}$ B1	6.1 ± 2.6	0.9 ± 0.5	6.6 ± 2.1	0.8 ± 0.4	2.3 ± 2.0	0.2 ± 0.1
E10	A1 $\xrightarrow []{}$ B2	60.9 ± 4.6	4027.6 ± 1904.8	54.1 ± 3.4	2531.6 ± 1341.1	65.2 ± 3.9	22933.6 ± 10923.1
E11	A2 $\xrightarrow []{}$ B1	1.7 ± 1.6	0.1 ± 0.2	4.5 ± 3.7	0.4 ± 0.5	4.6 ± 3.5	0.7 ± 0.9
E12	A2 $\xrightarrow []{}$ B2	8.5 ± 12.7	10.4 ± 30.1	18.7 ± 15.4	18.3 ± 29.3	63.1 ± 15.5	97874.2 ± 125079.4
E13	A3 $\xrightarrow []{}$ B1	4.4 ± 2.1	0.5 ± 0.3	6.9 ± 2.6	0.9 ± 0.6	4.4 ± 5.2	0.5 ± 0.7
E14	A3 $\xrightarrow []{}$ B2	13.7 ± 9.0	7.8 ± 11.6	23.9 ± 10.3	71.1 ± 141.4	44.2 ± 6.4	117.3 ± 85.6
E15	B1 $\xrightarrow []{}$ A1	32.7 ± 5.1	59.3 ± 86.9	41.5 ± 8.4	81.6 ± 70.7	45.8 ± 13.3	101.3 ± 86.4
E16	B1 $\xrightarrow []{}$ A2	32.6 ± 1.7	26.5 ± 7.8	36.3 ± 3.3	44.8 ± 23.0	36.2 ± 2.0	64.1 ± 21.5
E17	B1 $\xrightarrow []{}$ A3	21.6 ± 6.8	10.2 ± 8.6	7.7 ± 14.0	3.0 ± 6.2	5.2 ± 1.8	0.7 ± 0.2
E18	B2 $\xrightarrow []{}$ A1	30.2 ± 5.8	25.1 ± 16.0	31.9 ± 7.1	14.9 ± 6.0	34.8 ± 10.0	39.6 ± 18.9
E19	B2 $\xrightarrow []{}$ A2	35.5 ± 1.9	43.4 ± 13.4	35.8 ± 3.2	122.1 ± 179.7	47.0 ± 9.0	1510.6 ± 2003.2
E20	B2 $\xrightarrow []{}$ A3	1.5 ± 2.6	0.1 ± 0.3	10.7 ± 14.9	4.4 ± 8.0	6.1 ± 0.5	0.8 ± 0.1

It can be seen from Table 6 that compared with NoFPT-ADACNN and Kur-ADACNN, when the evaluation metric is RMSE, MD-ADACNN obtained the optimal value in 9 out of 12 cross-platform experiments (E11: A2 $\xrightarrow []{}$ B1, E12: A2 $\xrightarrow []{}$B2, E13: A3 $\xrightarrow []{}$ B1, E14: A3 $\xrightarrow []{}$B2, E15: B1 $\xrightarrow []{}$ A1, E16: B1 $\xrightarrow []{}$A2, E18: B2 $\xrightarrow []{}$ A1, E19: B2 $\xrightarrow []{}$A2, and E20: B2 $\xrightarrow []{}$ A3). When the evaluation metric is Score, MD-ADACNN obtained the optimal value in 8 of the 12 cross-platform experiments (E3: A2$\xrightarrow []{}$B1, E12: A2 $\xrightarrow []{}$ B2, E13: A3 $\xrightarrow []{}$ B1, E14: A3 $\xrightarrow []{}$B2, E15: B1 $\xrightarrow []{}$ A1, E16: B1 $\xrightarrow []{}$A2, E19: B2 $\xrightarrow []{}$ A2, and E20: B2 $\xrightarrow []{}$A3). We still find that the Kurtosis-based FPT detection method is unstable.

**Figure 6:**
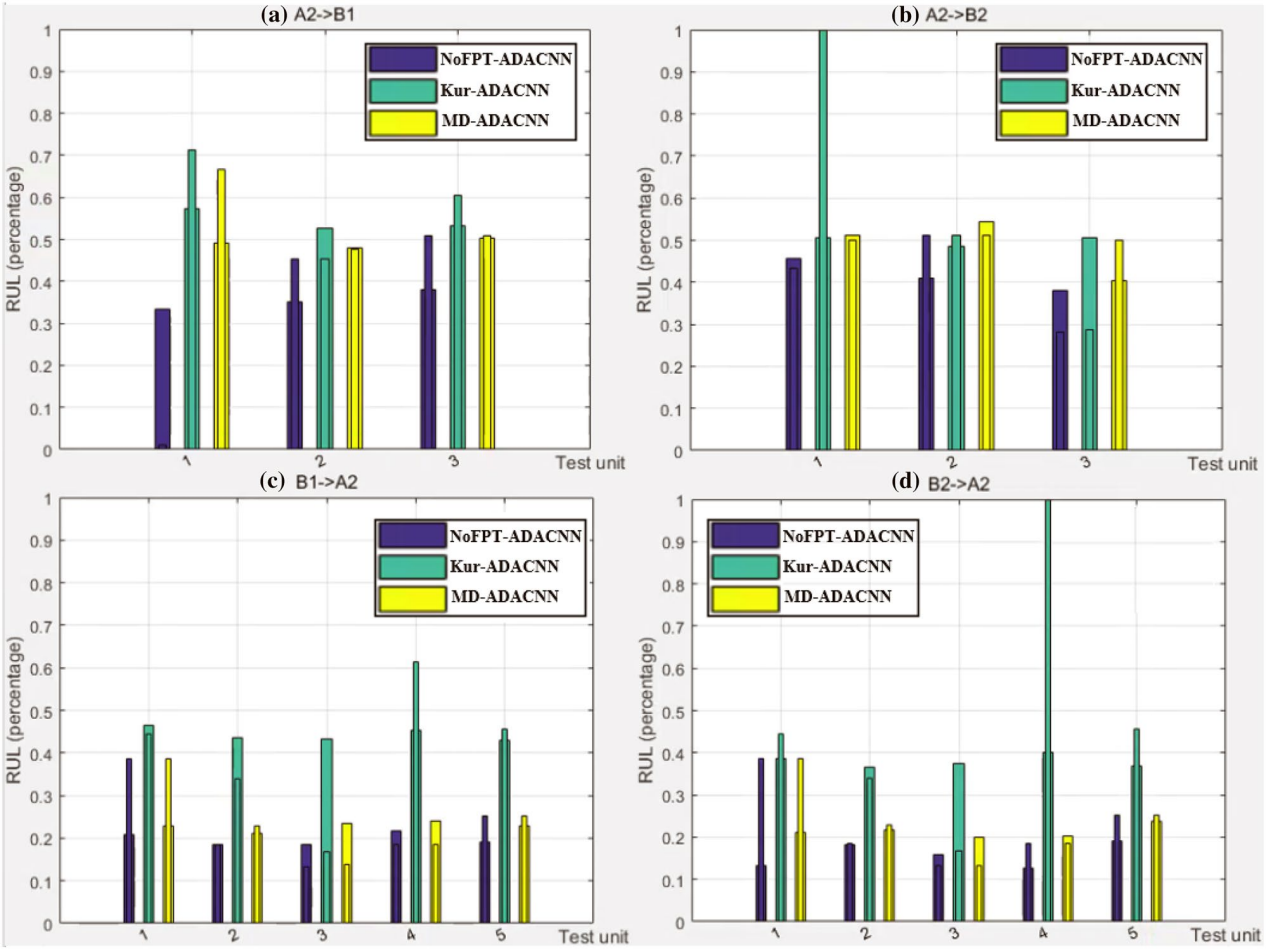
RUL estimation comparisons with different FPT detection mechanisms between two platforms: (**a**) E11: A2 $\xrightarrow []{}$ B1, (**b**) E12: A2 $\xrightarrow []{}$ B2, (**c**) E16: B1 $\xrightarrow []{}$ A2, (**d**) E19: B2 $\xrightarrow []{}$ A2.

It can be seen from Fig. 6 that in Fig. 6b,d, the Kur-based FPT detection mechanism considers the RUL label at the prediction point to be 1, while RUL label of the prediction point determined by the No FPT and MD-based FPT detection mechanism are different not big. From the principle that the majority obeys the minority, for test unit = 1 in Fig.6b and test unit = 4 in Fig.6d, we can think that Kur-based does not perform well. On the whole, whether it is A $\xrightarrow []{}$B or B$\xrightarrow []{}$A, the MD-ADACNN predicted value is not far from the corresponding label, and even more often slightly smaller than the corresponding label value.

### Feature visualization

**Figure 7:**
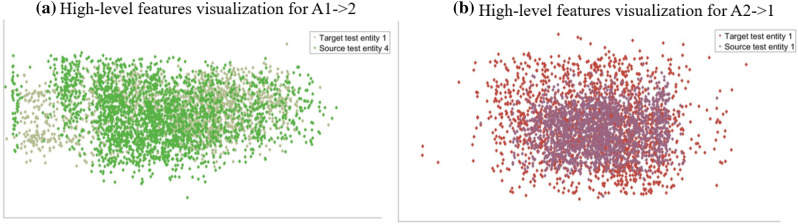
The feature visualization for cross-condition.

In order to demonstrate the effectiveness of the proposed model, we use the t-SNE method to visualize high-level features. Fig. 7 is the feature visualization in the case of knowledge transfer between different conditions under the same platform, that is, in Fig. 7a, the source domain test entity is based on condition 1 in FEMTO dataset, and the target test entity is based on condition 2 in FEMTO dataset, and in Fig. 7b, the source domain test entity is based on condition 2 in FEMTO dataset, and the target test entity is based on condition 1 in FEMTO dataset. From Fig. 7 we can see that the high-level features on the subspace corresponding to the source domain test entity data and the target domain test entity data are fully fused, which proves that the proposed domain adaptation method has played a role.

**Figure 8:**
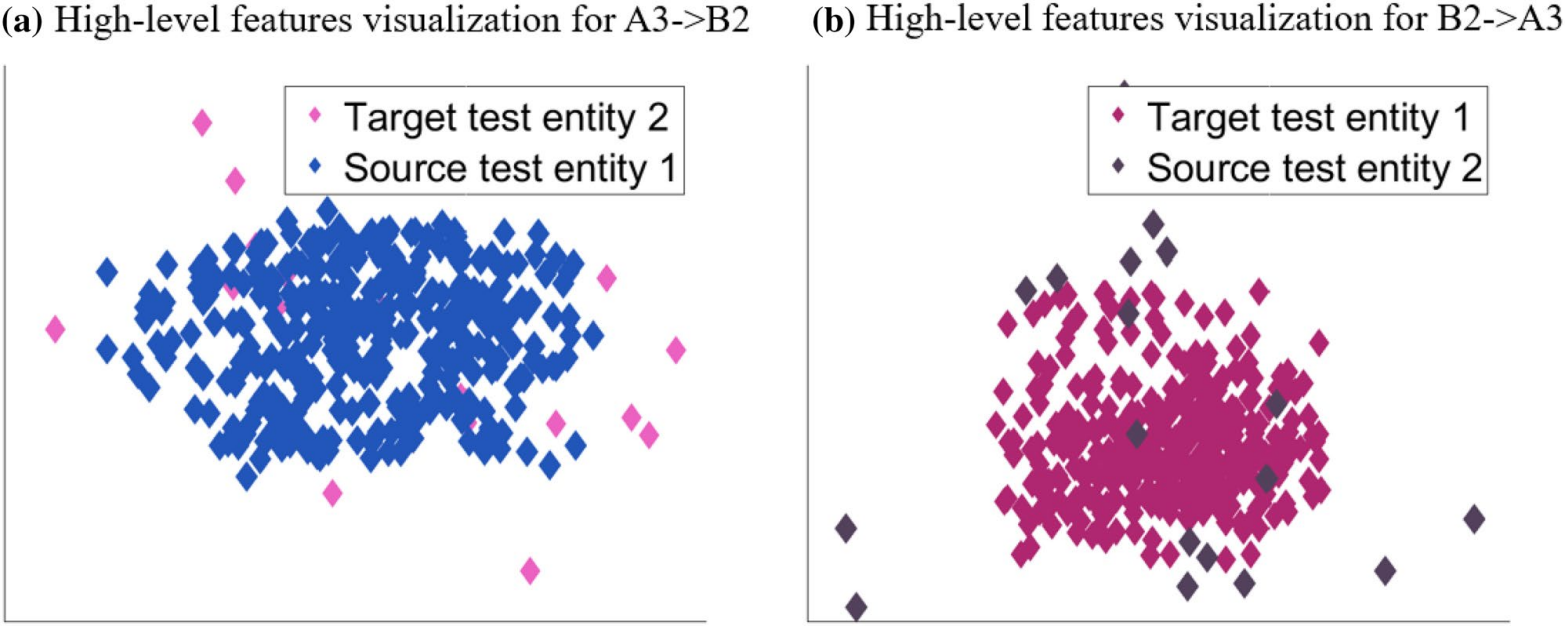
The feature visualization for cross-platform.

Figure 8 describes feature visualization for cross-platform. In Fig. 8a, the source data is condition 3 based on platform a, and the target data is condition 2 based on platform b. In Fig. 8b, the source data is condition 2 based on platform a, and the target data is condition 3 based on platform b. In the above two cases, it has big bridge between source test data and target test data. Because this research background is cross-platform and cross-condition, and the source domain condition is single, the training data information is not rich, so for the remaining life prediction task, the source domain knowledge is transferred to the target domain is quite difficult. Despite this, we can see from Fig. 8 that both the high-level features corresponding to source domain data and target domain data have a large overlap area to some extent.

Due to the difficulty in predicting the remaining life of cross-platform migration, such as different operating conditions, different sampling frequencies, and large differences in life span. In short, compared with Fig. 7, the monotonicity of Fig. 8 is not so obvious, but this does not mean that it is a complete failure. We can understand that such feature representation is a greater challenge for the subsequent regression predictor.
